# Supplementary material for: Incorporation of Hybrid Nanomaterial in Dental Porcelains: Antimicrobial, Chemical, and Mechanical Properties
Source: Antibiotics (Basel). 2021 Jan 20;10(2):98. doi: 10.3390/antibiotics10020098 (PMC7909241; doi:10.3390/antibiotics10020098)
Supplement: Supplementary file 1 [file antibiotics-10-00098-s001.pdf]

antibiotics

Article

Incorporation of Hybrid Nanomaterial in Dental Porcelains: Antimicrobial, Chemical, and Mechanical Properties

Carla L. Vidal, Izabela Ferreira, Paulo S. Ferreira, Mariana L. C. Valente, Ana B. V. Teixeira and Andréa C. Reis *

Dental Materials and Prosthesis Department, Ribeirão Preto School of Dentistry, University of São Paulo, Av. do Café, s/n, Ribeirão Preto 14040-904, Brazil; carla.vidal@usp.br (C.L.V.); izabela.ferreira@usp.br (I.F.); b14vilela@hotmail.com (P.S.F.); mariana.lima.valente@usp.br (M.L.C.V.); ana.beatriz.teixeira@usp.br (A.B.V.T.)
* Correspondence: andreare73@yahoo.com.br; Tel.: +55-16-3315-0477

Table 1. Chemical elements detected in commercial dental porcelains incorporated with β-$AgVO_3$ using energy-dispersive X-ray spectroscopy (EDS) (wt.%).

Chemical Element	IPS Inline			Ex-3 Noritake		
	control	2.5%	5%	control	2.5%	5%
C	0.00	0.00	0.00	0.00	0.00	0.00
O	45.46	44.47	44.02	44.88	42.79	43.88
Al	8.51	8.71	8.08	8.14	7.95	7.87
Si	29.20	29.48	28.33	29.16	31.87	29.07
P	0.00	0.00	0.00	0.00	0.02	0.00
S	0.00	0.00	0.00	0.00	0.02	0.00
Cl	0.02	0.00	0.00	0.03	0.00	0.08
K	8.93	8.73	8.65	7.40	6.53	6.98
Ca	0.86	0.83	0.81	0.56	0.69	0.57
Fe	0.05	0.08	0.05	0.04	0.02	0.04
Zn	6.73	7.02	6.90	9.73	9.01	9.07
Ag	0.25	0.40	2.40	0.05	0.53	1.78
V	0.00	0.27	0.75	0.00	0.58	0.64
